# The way to a design space for an animal cell culture process according to Quality by Design (QbD)

**DOI:** 10.1186/1753-6561-5-S8-P12

**Published:** 2011-11-22

**Authors:** Robert Puskeiler, Jan Kreuzmann, Caroline Schuster, Katharina Didzus, Nicole Bartsch, Christian Hakemeyer, Heike Schmidt, Melanie Jacobs, Stefan Wolf

**Affiliations:** 1 Roche Diagnostics GmbH, Pharma Biotech, Development Fermentation, Penzberg, Germany, 82377; 2 Roche Diagnostics GmbH, Pharma Research and Early Development Cell Sciences, Penzberg, Germany, 82377; 3 Roche Diagnostics GmbH, Pharma Biotech, Manufacturing Fermentation, Penzberg, Germany, 82377

## Background

The strategy of implementation of the QbD (Quality by design) approach in upstream processing of therapeutic proteins consists of the identification of critical process parameters (CPPs) that have a statistically significant influence on the critical quality attributes (CQAs) of a specific process. By applying the acceptance criteria to the CQAs, proven acceptable ranges (PARs) for the CPPs can be deduced from experimental data. The multidimensional combination of these ranges form the design space and thus assures the quality of the product.

The QbD approach according to the ICH guidelines Q8, Q9 and Q10 may be subdivided in the work packages scale down model qualification, risk analysis, process characterization and range studies. The foundation of the QbD approach is represented by the scale down model. Several different scale down criteria were applied and adapted until a satisfactory match of scale down to commercial scale data was achieved. The scale down model is then used to investigate cause effect relationships between process parameters and quality attributes of the production process.

Since a standard cell culture process from thawing of the vial up to the final production fermenter can comprise up to 100 process parameters, a risk based approach is helpful to filter the most important ones. Those parameters are then experimentally investigated to verify their criticality for the quality attributes of the process. This approach relies on design of experiment (DoE) to reduce the number of required experiments to a manageable number while maintaining meaningful results. During the range studies, those critical parameters will be investigated with the help of a high resolution DoE matrix in order to be able to reveal possible interactions and higher order effects.

## Scale down model

Based on development data a scale down model at 2 L scale was established. Predefined scale down criteria (power input, volumetric aeration rate, tip speed) were applied while taking the specific clone properties into account. The qualification of the scale down model was carried out by considering an acceptance criteria for several critical quality attributes and key performance indicators for at least three scale down fermentation runs. The acceptance criteria consisted of matching the 2-fold standard deviation range with the mean of the small scale data and the 3-fold standard deviation range with individual data points.

## Risk analysis

Being confronted with a large number of process parameters, risk assessment tools are used to focus the experimental efforts on the most relevant parameters. A Failure Mode and Effects Analysis (FMEA) based tool was applied to rate hypothetical deviations of a parameter from a previously defined observation range. The hypothetical deviation is rated by its severity, occurrence and detectability yielding a ranking of all parameters according to their risk priority number.

## Process characterization / range studies

**Figure 1:**
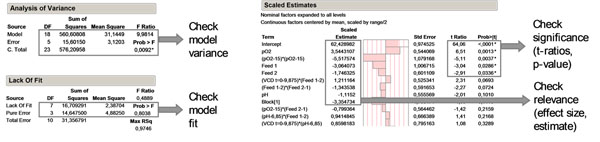
Statistical data evaluation from process characterization data. Models with satisfying ANOVA data and lack of fit were created to evaluate all significant and relevant process parameters. Plots created with JMP®, SAS, Cary, USA. DF = degrees of freedom, C. Total = total sum of squares, Prob> |t|= probability of getting a higher t-Ratio by chance (p-value).

The experimental investigation was started with a first round fractional factorial screening design with the parameters filtered by the risk analysis tool. The resulting models were optimized such that model quality was sufficient (p < 0.05) and no lack of fit was observed. The parameters were evaluated in terms of statistical significance by the t-ratio and p-value. Relevance of certain parameters was checked by comparison of the estimate size to the center point variation (Figure 1).

The results of that work package allowed to eliminate some process parameters from further experimentation due to their lack of significance and/or relevance. The resulting parameters were investigated in a second round of multivariate experimentation with a central composite design aiming at the definition of the unit operation design space. The higher resolution models were then subsequently used to define the design space mathematically. To that aim, each quality attribute was evaluated with its own optimized model only covering significant terms.

## Design space visualization

**Figure 2:**
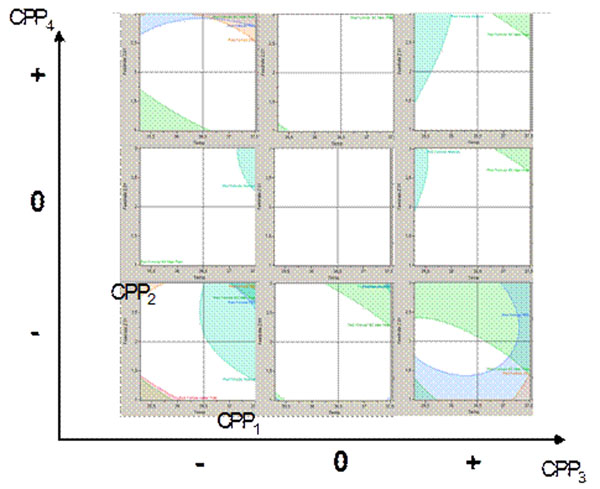
Example of the visualization of a design space for 4 parameters by the combination of 2 internal and 2 external axes.

The mathematical definition of the design space can, for example, be transformed into the following graphical representation (Fig. 2). Herein, the combination of two external and two internal parameter axes allow the visualization of 4 parameters in 9 two-dimensional plots. Every single plot depicts the proven acceptable range in white restricted by the mathematical models represented by the colored shaded areas. The models are derived from the experimental results and thus cut those areas that represent exceeded acceptance criteria.

A fully representative view of the 4-dimensional design space can thus be achieved if all possible permutations of the external and internal axes is shown.

